# Fatal Adverse Event After VLA1553 Chikungunya Vaccination in an Elderly Patient: A Case Report From Reunion Island

**DOI:** 10.1093/ofid/ofaf550

**Published:** 2025-09-03

**Authors:** Emilie Mosnier, Marie-Christine Jaffar-Bandjee, Radj Cally, Lotfi Dahmane, Etienne Frumence, Liem Binh Luong Nguyen, Rodolphe Manaquin, Muriel Vincent, Marie Pierre Moiton, Patrick Gérardin, Xavier de Lamballerie, Julien Jabot

**Affiliations:** Infectious and Tropical Diseases Unit, University Hospital of La Réunion, La Réunion, St Denis and St Pierre, France; INSERM, IRD, SESSTIM, Sciences Economiques & Sociales de la Santé & Traitement de L’Information Médicale, Aix Marseille Institute of Public Health ISSPAM, Aix Marseille University, Marseille, France; Infectious and Tropical Diseases Unit, University Hospital of La Réunion, La Réunion, St Denis and St Pierre, France; Microbiology/Virology Laboratory, Associated National Reference Centre for Arboviruses Reunion, University Hospital of La Réunion, St Denis, La Réunion, France; Intensive Care Unit, INSERM CIC1410, University Hospital of La Réunion, Saint Denis, Reunion Island, France; Infectious and Tropical Diseases Unit, University Hospital of La Réunion, La Réunion, St Denis and St Pierre, France; Microbiology/Virology Laboratory, Associated National Reference Centre for Arboviruses Reunion, University Hospital of La Réunion, St Denis, La Réunion, France; Infectious and Tropical Diseases Unit, University Hospital of La Réunion, La Réunion, St Denis and St Pierre, France; Inserm, CIC 1417, Hôpital Cochin, AP-HP, Paris, France; Plateforme de Recherche Clinique et Translationnelle, INSERM CIC1410, University Hospital of La Réunion, Saint-Pierre, La Réunion, France; Infectious and Tropical Diseases Unit, University Hospital of La Réunion, La Réunion, St Denis and St Pierre, France; Santé Publique France, Saint Denis, La Réunion, France; Infectious and Tropical Diseases Unit, University Hospital of La Réunion, La Réunion, St Denis and St Pierre, France; Infectious and Tropical Diseases Unit, University Hospital of La Réunion, La Réunion, St Denis and St Pierre, France; Plateforme de Recherche Clinique et Translationnelle, INSERM CIC1410, University Hospital of La Réunion, Saint-Pierre, La Réunion, France; Infectious and Tropical Diseases Unit, University Hospital of La Réunion, La Réunion, St Denis and St Pierre, France; Unité des Virus Émergents (UVE) and National Reference Centre for Arboviruses, Aix-Marseille Univ, IRD 190, INSERM 1207, Marseille, France; Infectious and Tropical Diseases Unit, University Hospital of La Réunion, La Réunion, St Denis and St Pierre, France; Intensive Care Unit, INSERM CIC1410, University Hospital of La Réunion, Saint Denis, Reunion Island, France

**Keywords:** chikungunya virus, encephalitis, live-attenuated vaccine, older adults, vaccine safety

## Abstract

We report a fatal case of febrile encephalopathy in an 84-year-old man following administration of the live-attenuated chikungunya vaccine IXCHIQ® (VLA1553, Valneva SE) during the 2025 outbreak in Réunion Island. The patient, previously autonomous with stable comorbidities, developed fever, asthenia, and polyarthralgia 3 days post-vaccination. His condition rapidly deteriorated, with confusion, acute kidney injury, and hemodynamic instability requiring intensive care. Chikungunya virus RNA was detected in both serum and cerebrospinal fluid, with sequencing confirming the presence of the vaccine strain in both compartments. Despite broad-spectrum antimicrobials, antiviral therapy targeting herpesviruses, and hemodialysis, his neurological status worsened and he died 14 days after symptom onset. This case is the first fatal adverse event officially recognized by French health authorities as plausibly related to VLA1553 vaccine. It raises concerns regarding potential neuroinvasive disease following vaccination in elderly individuals and highlights the importance of close clinical and virological evaluation in post-vaccination adverse events during outbreak settings.

Chikungunya virus (CHIKV) is an emerging alphavirus transmitted by *Aedes* mosquitoes, responsible for an acute febrile illness with polyarthralgia, and in some patients a debilitating post-chikungunya viral arthritis disease [1]. Chikungunya virus infection may also lead to severe systemic manifestations, including neurological complications, hepatitis, and myocarditis [2]. Whilst conventional vector control measures have been proved insufficient, vaccination—particularly to prevent severe forms in at-risk adults—became a strategic pillar for outbreak containment [3]. VLA1553 (IXCHIQ®, developed by Valneva, France) is a live-attenuated, single-dose chikungunya vaccine derived from the LR2006-OPY1 strain isolated during the 2005 Reunion epidemic [4], which received FDA approval in November 2023 and EMA authorization in July 2024, becoming the first licensed vaccine against chikungunya. Licensure was granted through a phase III study trial, which report immunogenicity data in 266 participants and safety data in 4115 participants. It showed nearly 99% vaccinated adults developing neutralizing antibodies maintained for up to 2 years [5, 6]. However, authorization was based on immunogenicity endpoints, without demonstration of real-world vaccine effectiveness, and data in high-risk populations, such as older adults with comorbidities, remained limited: in the VLA1553 safety cohort, median age was 45.0 (IQR: 32.0–57.0), and 346 participants aged 65 + were included, of whom only 59 were aged 75 years or older [5].

After a major outbreak due to an East-Central-South African (ECSA)-divergent clade, known as the Indian Ocean lineage in 2005–2006 [7], Reunion Island remained free from major transmission for over 2 decade, until a new epidemic emerged in August 2024, rapidly affecting all the island [8]. Following national French recommendations [9], a vaccination campaign free of charge was launched, prioritizing individuals aged 65 years and older—given the strong association between age and mortality during the 2006 epidemic [2, 7]—and secondarily targeting adults aged 18 and over with comorbidities. The population at increased risk for severe disease—including older adults and individuals with chronic health conditions—was estimated at 180 000 out of a total population of approximately 870 000 [10]. As of 25 April 2025, around 6418 doses of the VLA1553 vaccine had been administered on Reunion Island.

In this context, we report the first fatal case of post-vaccination encephalitis and acute renal failure temporally associated with VLA1553 vaccine administration.

## CASE REPORT

A 84-year-old man, previously independent and living alone on Reunion Island, received the live-attenuated chikungunya vaccine VLA1553 (Valneva SE) on 4 April 2025. Three days later, he experienced asthenia, polyarthralgia, and low-grade fever. On April 8, persistent symptoms led him to consult his general practitioner, who prescribed paracetamol. His general condition worsened, culminating in a syncopal episode with prolonged ground-level fall and febrile confusion (40°C), prompting emergency department admission during the night of April 9–10.

His medical history included ischemic heart disease with a stented myocardial infarction in 2005 with preserved left ventricular ejection fraction, type 2 diabetes mellitus treated with oral antidiabetic drugs, well-controlled asthma, obstructive sleep apnea treated with nocturnal positive airway pressure, hiatal hernia, hypercholesterolemia, and bilateral cataracts. Chronic medications included amlodipine-valsartan, clopidogrel, metformin, esomeprazole, cholecalciferol, zolpidem, and *Serenoa repens* extract.

At presentation to the emergency department, the patient was febrile (40°C), confused (Glasgow Coma Scale score: 14), and reported diffuse joint pain. Laboratory tests revealed elevated C-reactive protein (CRP) at 269 mg/L. A full-body CT scan was unremarkable. Upper urinary tract infection was initially suspected, though urine culture was contaminated. Empirical intravenous cefotaxime was initiated.

**Figure 1.**
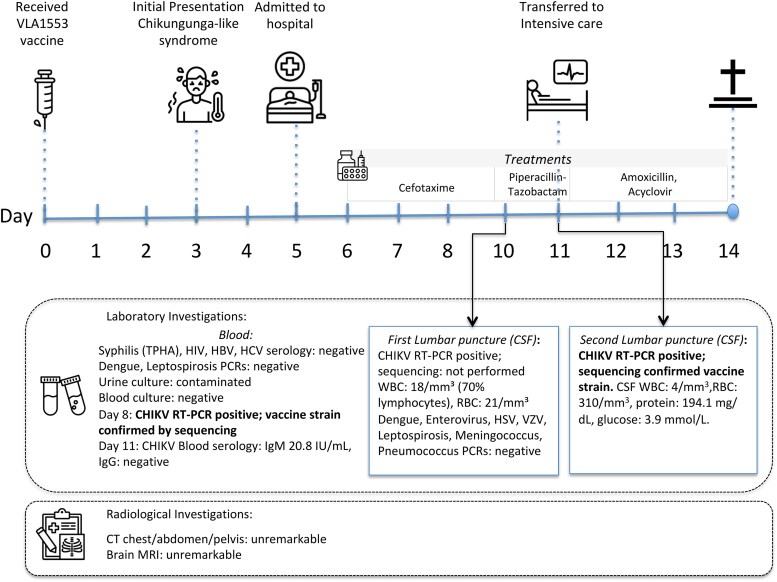
Timeline of clinical events, laboratory investigations, and imaging findings in a fatal case following VLA1553 vaccination.

On April 11, given worsening mental status and new-onset hemodynamic instability, the patient was transferred to the intermediate care unit, where he remained febrile (39.9°C) with altered mental consciousness (Glasgow score: 13). Neurologic examination revealed a horizontal gaze palsy suggestive of sixth nerve involvement, along with intermittent fasciculations, without myoclonus. Antimicrobial therapy was escalated to piperacillin-tazobactam and amikacin. On April 12, CHIK-V RNA was detected in plasma by RT-PCR (Figure 1).

Other systemic examinations were within normal limits, including cardiopulmonary and abdominal exams. He subsequently developed oliguria and worsening renal function, while echocardiography showed no systolic or diastolic dysfunction. Laboratory work-up revealed CRP at 252 mg/L, white blood cell count of 14 170/mm³, hyperglycemia (2.33 g/L), elevated troponin (122 ng/L), hepatic cytolysis (AST 218 IU/L, ALT 105 IU/L), and cholestasis (GGT 76 IU/L, ALP 100 IU/L).

On April 13, a lumbar puncture yielded clear cerebrospinal fluid with mild lymphocytic pleocytosis (18 leukocytes/mm³; 70% lymphocytes, 30% neutrophils) and 21 red blood cells/mm³, consistent with possible viral encephalitis. CHIKV RNA was detected in the CSF by RT-PCR (Ct: 27). Due to neurological deterioration, the patient was transferred to the intensive care unit (ICU) on April 14. Upon ICU admission, he was in atrial fibrillation and exhibited impaired consciousness without focal neurological deficit (Glasgow Coma Scale score fluctuating between 9 and 13). A repeat echocardiogram confirmed preserved cardiac function. Brain MRI was unremarkable.

At this stage, the patient met the International Encephalitis Consortium diagnostic criteria for possible encephalitis [11], which include: altered mental status lasting >24 hours with no alternative cause identified, plus CSF pleocytosis and a positive viral PCR.

On April 14, a second lumbar puncture was performed, and empiric acyclovir and high-dose amoxicillin were initiated, adjusted for renal function. The cerebrospinal fluid revealed 310 red blood cells/mm³ and 4 white blood cells/mm³. CHIKV RNA was detected on this second CSF specimen by RT-PCR (Ct: 28). Phylogenetic analysis of the viral genome confirmed 100% identity with the VLA1553 vaccine strain, clearly distinguishing it from the circulating epidemic strains in Réunion Island. Levetiracetam (Keppra) was introduced for convulsions (suspected partial seizures). Hemodialysis via femoral catheter was initiated on April 15 for acute kidney injury and suspected beta-lactam accumulation, but it did not lead to neurologic improvement.

On April 18, respiratory function and consciousness deteriorated to an unrousable coma with hypoventilation. The patient died later that day. Although EEG and MRI findings remained unremarkable, the clinical course strongly suggested severe viral encephalitis with possible brainstem involvement, leading to respiratory failure. The contribution of systemic complications such as renal dysfunction and metabolic disturbance could not be excluded, but neurologic deterioration was predominant. A decision not to initiate invasive mechanical ventilation, in agreement with the family and the medical team, was made, which contributed to the rapid fatal outcome.

## METHODS

### Serological Testing

Detection of anti-CHIKV IgM and IgG antibodies was performed using the Euroimmun anti-Chikungunya virus IgM and IgG ELISA kits (Euroimmun, Germany). The assays were carried out on the automated ETI-Max 3000 analyzer (Diasorin, Italy), according to the manufacturer's instructions.

### CHIKV RT-PCR

Viral RNA was extracted from 200 µl of patient plasma or CSF using the NucliSENS easyMAG kit on the easyMAG or eMAG automated systems (bioMérieux, France), following the generic extraction protocol recommended by the manufacturer. CHIKV detection was performed using an in-house one-step quantitative RT-PCR on the LightCycler 480 Real-Time PCR System (Roche, Switzerland), with the TaqMan Fast Virus 1-Step Master Mix (Applied Biosystems, USA). The assay used the following primers and probe targeting the CHIKV genome: CHIK-F AAGCTYCGCGTCCTTTACCAAG, CHIK-R CCAAATTGTCCYGGTCTTCTT, and CHIK-P 5′-FAM-CCAATGTCYTCMGCCTGGACACCTTT-BHQ1–3′ [12].

### Sequencing

Genome library preparation was performed directly from viral RNA extracted from positive samples using an in-house amplicon-based next-generation sequencing (NGS) protocol with custom CHIKV primer schemes on a MinION device (Oxford Nanopore Technologies, ONT), as previously described [13]. Raw sequencing data were basecalled using the latest super-accurate model, filtered for high-quality reads (mean Q-score >15), and demultiplexed using the MinKNOW software (ONT). Consensus genome sequences were then generated with the ARTIC network's field bioinformatics pipeline (version 1.6.1), using the CHIKV reference genome NC_004162. Genomes were aligned using Geneious Prime software (version 2025.1.1) with the MAFFT multiple sequence aligner (version 1.5.0), applying default parameters. Sequences were trimmed to remove free-end gaps, retaining only the coding sequence [14]. Automatic annotation was performed using the CHIKV reference genome.

Genomes were compared to the currently circulating epidemic strain in Réunion from the 2024–2025 outbreak (GenBank accession PV035814), the La Réunion 2006 strain OPY1 (GenBank accession KY575571), and the sequence of the live-attenuated vaccine VLA1553 [15].

Phylogenetic analyses were performed by constructing a maximum-likelihood tree using the PhyML tool (version 2.2.4) with 1000 bootstrap replicates.

### Ethics Statement

This case report was based on anonymized data collected during routine clinical care. In accordance with French regulations and the institutional policy of the University Hospital of Reunion Island, no additional ethical approval was required. The patient had not expressed opposition to the use of clinical data for research purposes.

## RESULTS

On 12 April 2025, plasma PCR for CHIKV returned positive, with a Ct value of 27.16. On April 14, CHIKV PCR in CSF was also positive (Ct 29.28). CHIKV serology performed the same day showed positive IgM (20.7 IU/mL) and negative IgG, consistent with an early-phase post-vaccination humoral response.

**Figure 2.**
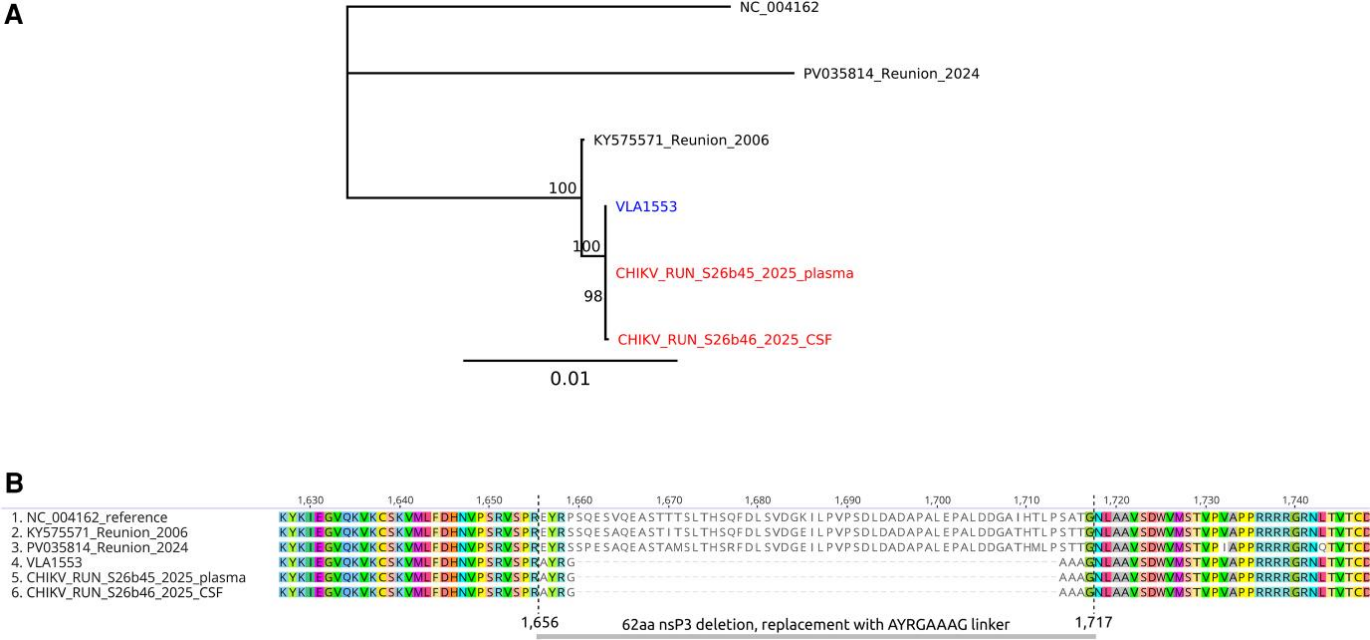
Genetic confirmation of vaccine-derived chikungunya virus in plasma and CSF samples. *A*, Maximum-likelihood phylogenetic tree comparing the patient's CHIKV sequences from plasma (CHIKV_RUN_S26b45_2025) and CSF (CHIKV_RUN_S26b46_2025) with the La Réunion 2006 strain, the 2024 circulating strain, and the VLA1553 strain. *B*, Amino acid alignment focusing on the region characteristic of the live-attenuated vaccine strain, highlighting the 62-amino-acid deletion in nsP3 (residues 1656–1717 of the nsP1234 polyprotein). Phylogenetic reconstruction and alignment visualization were performed using Geneious Prime (Biomatters Ltd., Auckland, New Zealand).

Sequencing of the samples performed using Oxford Nanopore NGS technology generated consensus sequences covering up to 88% of the reference genome at ≥100× coverage depth, with an average coverage depth exceeding 1000× for both samples. Phylogenetic analysis showed that two sequences clustered very closely with the VLA1553 strain and were distant from the currently circulating epidemic strain (Figure 2*A*). CHIKV RNA detected in plasma (April 12) and cerebrospinal fluid (April 14) samples was confirmed by sequencing to be 100% identical to the VLA1553 vaccine strain in all unambiguous regions. Only the sequence from the April 12 plasma sample is represented in the phylogenetic tree shown in Figure 1*A*.

In addition, sequence alignment analysis revealed a 62-amino-acid deletion in the nsP3 region (residues 1656–1717 of the nsP1234 polyprotein), which is a known and stable genetic marker introduced during attenuation and specific to the VLA1553 vaccine strain. The analysis also identified the linker sequence characteristic of this vaccine. These findings confirm the presence of the live-attenuated VLA1553 strain in the patient's plasma and cerebrospinal fluid (CSF) samples (Figure 2*B*).

In-depth analysis of the sequenced reads (BAM files) did not reveal the presence of the 2024–2025 Reunion strain in either of the sequenced samples.

## DISCUSSION

To our knowledge, this is the first reported case of a fatal serious adverse event (SAE) in temporal association with the VLA1553 live-attenuated chikungunya vaccine, for which the causal relationship appears plausible. The patient, an 84-year-old man with stable chronic comorbidities and preserved autonomy, developed a rapidly progressive febrile encephalopathy 5 days after vaccination. Chikungunya virus RNA was detected both in plasma and cerebrospinal fluid, and sequencing confirmed the presence of the vaccine strain VLA1553 in both compartments. The detection of vaccine-derived CHIKV in the central nervous system, along with an early humoral IgM response and the absence of other identifiable causes, strongly supports a vaccine-related pathophysiological process.

Severe manifestations of CHIKV infection at the acute phase of the disease, although historically considered rare, have been increasingly reported in recent outbreaks [16]. Data from La Réunion, the Caribbean, and Brazil highlight that severe cases can involve cardiovascular, neurological, and renal complications, particularly among elderly adults and individuals with comorbidities [7, 17, 18]. Hospitalization rates for severe forms range from 0.2% to 0.6% of infected individuals, with mortality estimates between 0.1% and 0.3% [16]. During the current outbreak, 9 deaths occurring between weeks 11 and 14 of this year in individuals over the age of 70 with underlying comorbidities have been classified as chikungunya-related—7 directly and 2 indirectly [10]. Several additional deaths are currently under investigation to assess a potential causal link with CHIKV infection. These fatalities must be interpreted in the context of a large-scale epidemic, with over 44 000 confirmed cases and more than 160 000 consultations for suspected CHIKV infection reported since the beginning of 2025 [10]. Encephalitis is the most commonly reported neurological complication associated with CHIKV infection and tends to affect primarily neonates, young infants, and elderly patients [19, 20]. These observations are consistent with the recognized neurotropism of CHIKV [21, 22], especially of East-Central-South African (ECSA) divergent Indian Ocean lineage—the origin of the live-attenuated strain used in VLA1553—which has been associated with neurological complications such as encephalitis, particularly in vulnerable populations [22]. Indeed, the incidence of CHIKV encephalitis recapitulates the known U-shaped pattern for infectious encephalitides which concerns infants less than 6 months and elderly adults of 65 years and more [23].

In pooled safety data from phase I and III trials involving 3610 participants, “chikungunya-like adverse reactions” were identified in 12.1% of vaccine recipients [5, 6, 24]. These reactions were broadly defined as fever in combination with at least one symptom typically seen in acute chikungunya virus infection, such as arthralgia, myalgia, rash, headache, or certain neurological, cardiac, or ocular manifestations. Most frequently reported combinations involved fever with headache, fatigue, myalgia, or arthralgia, while other symptoms occurred in fewer than 10% of cases. The median onset was 3 to 4 days post-vaccination, and most symptoms were mild and self-limited, with a median duration of four days.

Data on older adults remain limited. In the phase 3 trial, only 346 participants aged ≥65 years were included, and only 5 individuals aged ≥85 years were enrolled, precluding meaningful interpretation of safety in the oldest age group [5]. In this study, the VLA1553 vaccine was well tolerated in individuals aged ≥65 years, with no specific safety signals identified in this age group during the trial. However, as the FDA's Clinical Review Memo for the vaccine reports, while the incidence of solicited local and systemic adverse events was lower in older adults compared to younger participants, the rates of serious adverse events (3.5%) and medically attended adverse events (17.6%) were higher in those aged 65 years and older, compared to 1.2% and 11.8% in participants aged 18–64 [25]. This pattern is consistent with increased susceptibility to vaccine-related complications in older individuals, potentially driven by age-related immune dysfunction. Notably, 2 participants experienced serious adverse reactions requiring hospitalization: one, a 58-year-old woman with fibromyalgia, developed severe myalgia and cardiorespiratory symptoms; the other, a 66-year-old man, presented with severe fever, atrial fibrillation, elevated troponin and natriuretic peptide levels, and hyponatremia [5]. In both cases, symptoms began within days of vaccination. For the latter, vaccine-derived viremia was confirmed on day 7; while viremia is expected following administration of a live-attenuated vaccine, this finding raises the possibility of more substantial viral replication in certain susceptible individuals.

Post-licensure surveillance data from the United States have since reported 6 serious adverse events in elderly individuals following VLA1553 vaccination [26]. Among them, multiples cases of neurological involvement, including encephalopathy and encephalitis, have been described. For example, an 83-year-old man developed encephalopathy with persistent weakness after an episode of acute kidney injury and metabolic disturbance, and an 86-year-old man required prolonged intensive care for toxic metabolic encephalopathy associated with chikungunya viremia. Notably, one case of aseptic meningitis was also reported in a 68-year-old man with chikungunya-specific IgM and neutralizing antibodies identified in cerebrospinal fluid. These clinical presentations, temporally associated with vaccination (typically within 3–5 days), and in some instances supported by molecular confirmation of the vaccine strain, underscore the plausibility of vaccine-associated neuroinvasive complications in elderly individuals, as illustrated in the present case [27]. To date, no similar fatal or severe neuroinvasive adverse events have been reported in younger adults following VLA1553 vaccination, either in phase 3 clinical trials involving more than 4000 participants or in post-marketing pharmacovigilance data from the ANSM, EMA, or FDA [5, 24, 26, 28]. While such an event cannot be considered impossible, current evidence suggests that the risk is likely very low in this age group, highlighting the importance of maintaining active surveillance across all vaccinated populations.

The occurrence of serious adverse events shortly after VLA1553 vaccination in vulnerable individuals raises important pathophysiological hypotheses regarding both host-related and virus-related factors.

In this case, the patient had no specific identified cause of immunosuppression, but his advanced age was likely accompanied by immunosenescence [29], associated with a decline in both innate and adaptive immune defenses, which can compromise the response to viral infections and vaccines [27]. In addition, type I IFN is the key to prevent CHIKV dissemination to the central nervous system [30], the prevalence of autoantibodies neutralizing type I IFNs increases with age and such antibodies have been associated with severe encephalitic presentations in the case of West Nile virus, another arbovirus [31]. This specific item warrants further investigation. Altogether, the simultaneous presence of the vaccine strain genome in the blood and cerebrospinal fluid, as in our case, as well as the absence of specific mutations in the vaccine strain that could promote increased virulence, support the hypothesis of insufficiently controlled replication by the patient. Although the VLA1553 vaccine demonstrated an acceptable safety profile in younger healthy adults, it remains therefore possible that it retains a replicative capacity too high for safe use in individuals with reduced immune defenses. This concern is reminiscent of the yellow fever vaccine, another live-attenuated virus, which has been associated with a higher incidence of severe adverse events in older adults [32].

Our findings underscore the importance of carefully evaluating the safety of live-attenuated vaccines in elderly and highly comorbid populations, particularly during outbreak settings where rapid vaccination campaigns may prioritize vulnerable groups. Although VLA1553 provides a critical tool for chikungunya prevention, the occurrence of serious adverse events in vulnerable individuals—consistent with emerging post-marketing data in the United States—suggests the need for targeted risk mitigation strategies.

Among the 3 fatal cases reported in elderly individuals following VLA1553 vaccination in mainland France and Reunion Island, this case is the only one for which the French health authorities (ANSM) considered a causal link with the vaccine to be very likely, based on the chronology, clinical presentation, and biological findings. For the 2 other cases, no causal link could be established [28].

Active pharmacovigilance, enhanced post-vaccination monitoring, and clear risk stratification protocols should be systematically implemented, especially in individuals aged ≥65 years with comorbidities. Further research is urgently needed to elucidate the mechanisms underlying severe vaccine-related adverse events in this population and to refine vaccine indications accordingly.

Until more definitive data are available, a cautious, individualized approach to VLA1553 vaccination in older adults appears warranted to balance outbreak control with patient safety. In light of these concerns, on 26 April 2025, French health authorities suspended VLA1553 vaccination for individuals over 65, in line with EMA PRAC-ETF recommendations to exercise caution in frail older adults [33, 34]. The EMA-approved VLP vaccine V181 (VIMKUNYA®, Bavarian Nordic), being non-replicating, could be considered as an alternative for this population, although data in the elderly remain limited [35].
